# Low CO_2_ concentration, a key environmental factor for developing plateau adapted rapeseed

**DOI:** 10.1186/s13068-024-02481-w

**Published:** 2024-02-21

**Authors:** Sha Liu, Lin Tang, Jingyan Fu, Caixia Zhao, Ying Zhang, Meng Yin, Maolin Wang, Rui Wang, Yun Zhao

**Affiliations:** 1 Key Laboratory for Bio-Resources and Eco-Environment, College of Life Sciences, Sichuan University, Chengdu, China; 2 Tibet Academy of Agriculture and Animal Husbandry Sciences, Lhasa, China; 3 Science and Technology Innovation Center of Sichuan Modern Seed Industry Group, Chengdu, China

**Keywords:** Rapeseed, Plateau, Low carbon dioxide, Photosynthesis, Leaf carbon metabolism

## Abstract

**Background:**

Photosynthesis is a fundamental process that underlies the formation of crop yield, wherein light serves as the driving force and carbon dioxide (CO_2_) as the raw material. These two factors have a direct influence on the progress and efficiency of photosynthesis in crops. Rapeseed is one of the four major oilseed crops worldwide. Plateau rapeseed has now become a research hotspot. However, the lack of high-yielding rapeseed germplasm resources on the plateau and the highly efficient strategy for screening them severely affect the development of rapeseed industry in plateau.

**Results:**

In the rapeseed experimental fields located on the plateau (Lhasa, Tibet), we measured abundant sunlight, characterized by an average daily photosynthetically active radiation (PAR) of 1413 μmol m^−2^ s^−1^. In addition, the atmospheric CO_2_ concentrations range from 300 to 400 ppm, which is only two-thirds of that in the plain (Chengdu, Sichuan). We found that under different measurement conditions of light intensity and CO_2_ concentration, different rapeseed genotypes showed significant differences in leaf photosynthetic efficiency during the seedling stage. Moreover, the rapeseed materials with high photosynthetic efficiency under low CO_2_ concentrations rather than high light intensity, exhibited significant advantages in biomass, yield, and oil content when cultivated on the plateau, indicating that the CO_2_ is the key environmental factor which limited rapeseed production in plateau. Based on photosynthetic efficiency screening under low CO_2_ concentrations, six rapeseed varieties SC3, SC10, SC25, SC27, SC29 and SC37, shown significantly higher yields in plateau environment compared to local control variety were obtained. In addition, the adaptability of rapeseed to plateau was found to be related to the activities of key Calvin cycle enzymes and the accumulation of photosynthetic products.

**Conclusions:**

This study established a screening strategy for plateau high-yielding rapeseed materials, obtained six varieties which were suitable for plateau cultivation, explored the mechanism of rapeseed response to the plateau environment, and thus provides a feasible strategy for plateau-adapted rapeseed breeding.

**Supplementary Information:**

The online version contains supplementary material available at 10.1186/s13068-024-02481-w.

## Background

**Fig. 1:**
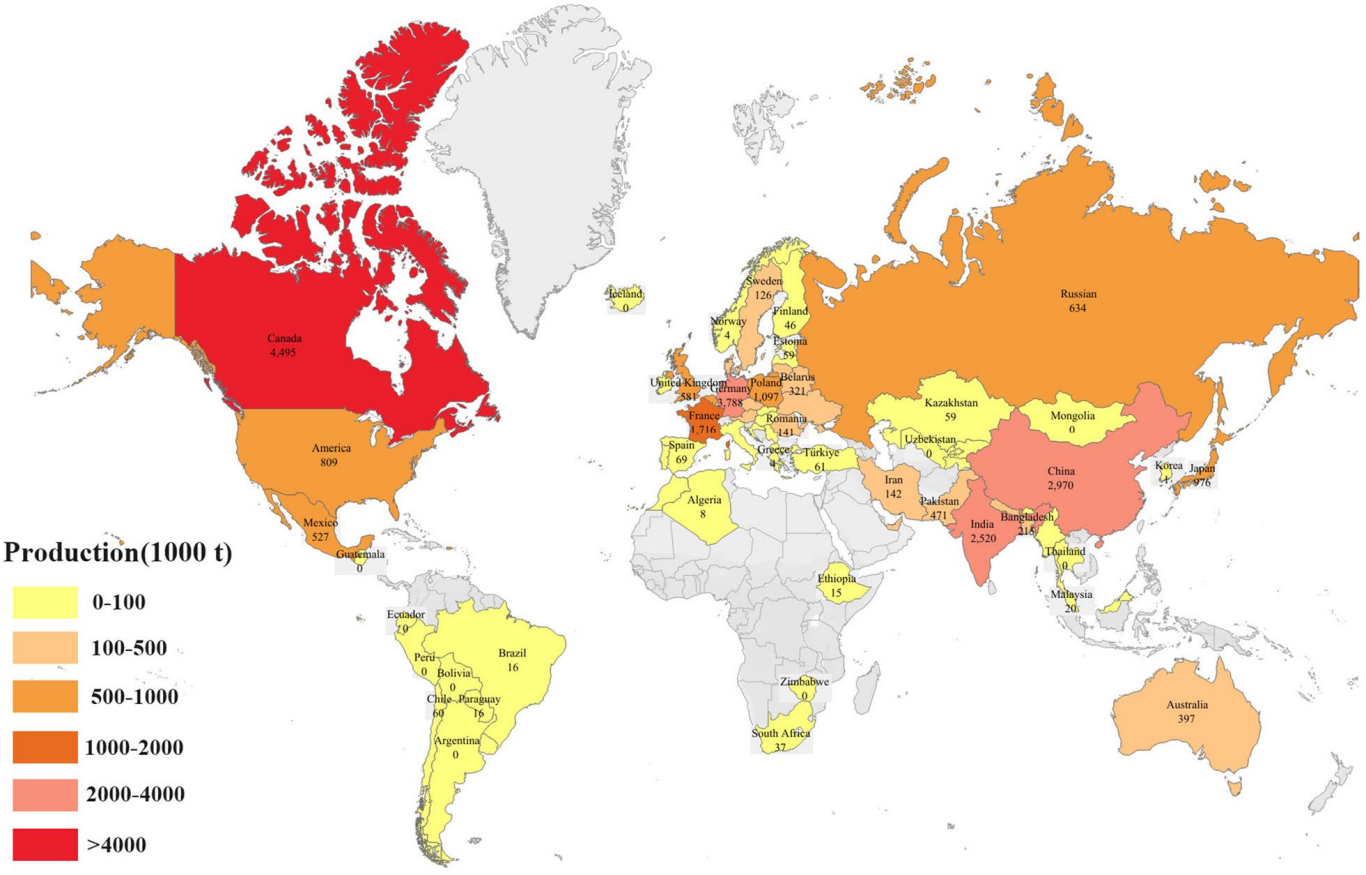
Distribution and production of rapeseed in the world in 2021, with data from the Food and Agriculture Organization of the United Nations (FAO)

As the world’s fourth largest oilseed crop, rapeseed is versatile and serves as an important source of edible oil, industrial fuel and feed [1]. Rapeseed is widely cultivated worldwide and is a key component of the agricultural system in many countries. China, Canada, India, European countries (particularly France, Germany and the United Kingdom) and the United States are the major producers of oilseed rape (Fig. 1). Demand for rapeseed is increasing as the world's population continues to grow, arable land continues to decline and the energy needs of industrial development continue to rise. However, the growth and yield of rapeseed is easily affected by climate and environmental conditions. The low-altitude plains have a mild climate suitable for large-scale rapeseed cultivation, and the terrain is flat, which facilitates mechanized harvesting. However, in the plateau region, the rapeseed industry faces the lack of germplasm resources, a low level of mechanization, and an urgent need to increase yield and quality due to the unique climatic ecological environment [2]. Since the plateau region has a vast land area and abundant sunlight resources, effective use of the potential agricultural resources of the plateau region, selection and breeding of new high-yielding rapeseed varieties suitable for growing on the plateau is crucial for improving global oilseed rape production, promoting the development of global agricultural production, and ensuring food and oil security.

Photosynthesis serves as the physiological foundation for crop yield formation, with 90–95% of a plant's dry matter directly from photosynthesis [3]. In recent years, numerous studies have shown that photosynthetic efficiency, a key factor influencing crop growth and yield, exerts a significant impact across various crops, environmental conditions, and growth stages. The variances in leaf photosynthetic effectiveness may account for crop yield disparities observed among different varieties [4], with high photosynthetic efficiency crops having an obvious yield advantage [5]. It has been reported that biotechnology-based enhancements of light protection mechanisms in tobacco resulted in an increased leaf photosynthesis rate, leading to a notable 15% rise in biomass yield [6]. The increase in wheat yield is associated with higher leaf photosynthesis rate and stomatal conductance [7]. In addition, decreased yield of rice [8], maize [9], and potatoes [10] has been reported as a consequence of covariantized photosynthesis under stress conditions. Net photosynthetic rate of different rapeseed genotypes was either significantly different or positively correlated with the yield per plant at all reproductive stages [11]. Therefore, enhancing rapeseed's light energy utilization and selecting high photosynthetic efficiency germplasm resources are crucial for boosting rapeseed yield in the plateau region, building on existing agricultural practices.

The efficiency of photosynthesis in crops is influenced by several factors, with light intensity and carbon dioxide concentration being the important environmental factors affecting photosynthesis. Studies have shown that low light intensity has a significant negative impact on soybean leaf weight, stomatal density, and photosynthesis [12]. Low light intensity conditions limit the synthesis of photosynthetic products in the whole growth period of rice, leading to yield reduction [13]. C_3_ plant leaves reach saturation at the light intensity of 800–1600 µmol m^−2^ s^−1^, meaning that the photosynthetic rate no longer increases with increasing light intensity [14]. CO_2_ is the raw material for plant photosynthesis. When the CO_2_ concentration reaches 700 ppm the photosynthetic rate of radish leaves increases by 20–28% compared to 400 ppm, with a dry weight increase of approximately 27% [15]. Elevated CO_2_ concentration significantly increases wheat photosynthetic rate, stomatal resistance, and water use efficiency [16].

Enzyme activity is also a key factor limiting crop photosynthetic efficiency. Rubisco catalyzes the fixation of CO_2_. However, Rubisco is a bifunctional enzyme, involved in both carboxylation of CO_2_ and photorespiration [17]. Therefore, the activity of ribulose-1,5-bisphosphate carboxylase (RuBPcase) has a limiting effect on carbon assimilation in C_3_ plants. Moreover, Rubisco needs to be activated by Rubisco activase (RCA) to function. Increasing the content of RCA without reducing Rubisco content can increase rice yield under high-temperature stress [18]. Sedoheptulose-1,7-bisphosphatase (SBPase) is involved in the regeneration of RuBP. Increased SBPase activity provides advantages in terms of carbon fixation and tolerance to oxidative stress induced by chilling in tomato plants [19]. Co-expression of SBPase and Rubisco can improve photosynthetic rate in rice [20]. Carbonic anhydrase (CA) catalysis the reversible reaction between CO_2_ and HCO_3_^−^, reducing the diffusion resistance of CO_2_ in the chloroplasts and providing the substrate CO_2_ for the carboxylation reaction, which affects the magnitude of the photosynthetic rate of the plant [21]. Phosphoenolpyruvate carboxylase (PEPCase) fills the tricarboxylic acid cycle by supplementing oxaloacetate in C_3_ plants, is involved in the synthesis of organic acids, and is associated with the remobilization of CO_2_ [22]. Meanwhile, photosynthesis is the major carbon metabolic pathway in plants, converting CO_2_ from the atmosphere and H_2_O into organic compounds, providing energy and a carbon source for plants [23]. Soluble sugars and starch are the main forms of carbon storage in plants. Increasing carbohydrate production and recycling in source organs is an important measure for achieving high crop yields [24]. Photosynthesis not only affects carbohydrate synthesis but also impacts other pathways related to carbon metabolism. For example, the supply of photosynthetic products can regulate nitrogen metabolism, lipid metabolism, and more.

At present, with global climate warming and an increasing greenhouse effect, most studies mainly focus on the relationship between elevated CO_2_ concentration and crop yield, neglecting the unique ecological conditions in plateau regions with low atmospheric CO_2_ concentration, which also affect crop yield. Taking the Qinghai–Tibet Plateau and the Western Sichuan Plateau in China as examples, the average annual solar radiation in these regions is about two to three times that of the same latitude [25]. While the atmospheric CO_2_ concentration in these plateau regions is about 300 ppm [26], regions at the same latitude but lower altitude have reached 412 ppm, showing a yearly increasing trend [27]. In addition, the plateau region has a complex terrain, inconvenient transportation and low labor. Within the limited agricultural production time, people prefer to cultivate food crops, resulting in the unbalanced development of food and oil in the plateau region and the slow development of the rapeseed industry. Therefore, it is both practically and theoretically significant to study the key factors limiting yield improvement of rapeseed in the plateau region. This includes enhancing plant breeding for these limiting factors, shortening the rapeseed breeding period in the plateau, and establishing an effective screening system for advantageous rapeseed varieties. The aim is to create new materials with high photosynthetic efficiency and high-yield rapeseed in the plateau.

This study compared environmental factors in rapeseed cultivation areas on the Tibetan Plateau and the Chengdu Plain to explore non-biological factors that may limit rapeseed yield improvement on the plateau. We compared the differences in photosynthetic rate among 40 rapeseed (*Brassica napus* L.) varieties under different measurement conditions and identified rapeseed varieties with high photosynthetic efficiency under various conditions. Furthermore, by integrating the differences in photosynthetic parameters, growth status, agronomic traits, and seed quality of high-photosynthesis genotypes grown in the Tibetan region, we elucidated the key environmental factor affecting rapeseed production on the plateau. We successfully obtained new high photosynthetic rate and high-yield rapeseed genotypes suitable for plateau conditions. In addition, by analyzing and comparing changes in the activity of key enzymes in the Calvin cycle, photosynthetic product content, and seed fatty acid composition in rapeseed under plateau environments, we delved into the molecular mechanisms through which high-photosynthesis genotypes of rapeseed respond to the unique ecological environment of the plateau. Our research aims to establish methods for optimizing the photosynthesis process to increase yield, thereby contributing to edible oil security and sustainable agriculture in the plateau regions.

## Results

### Differences in environmental factors

**Fig. 2:**
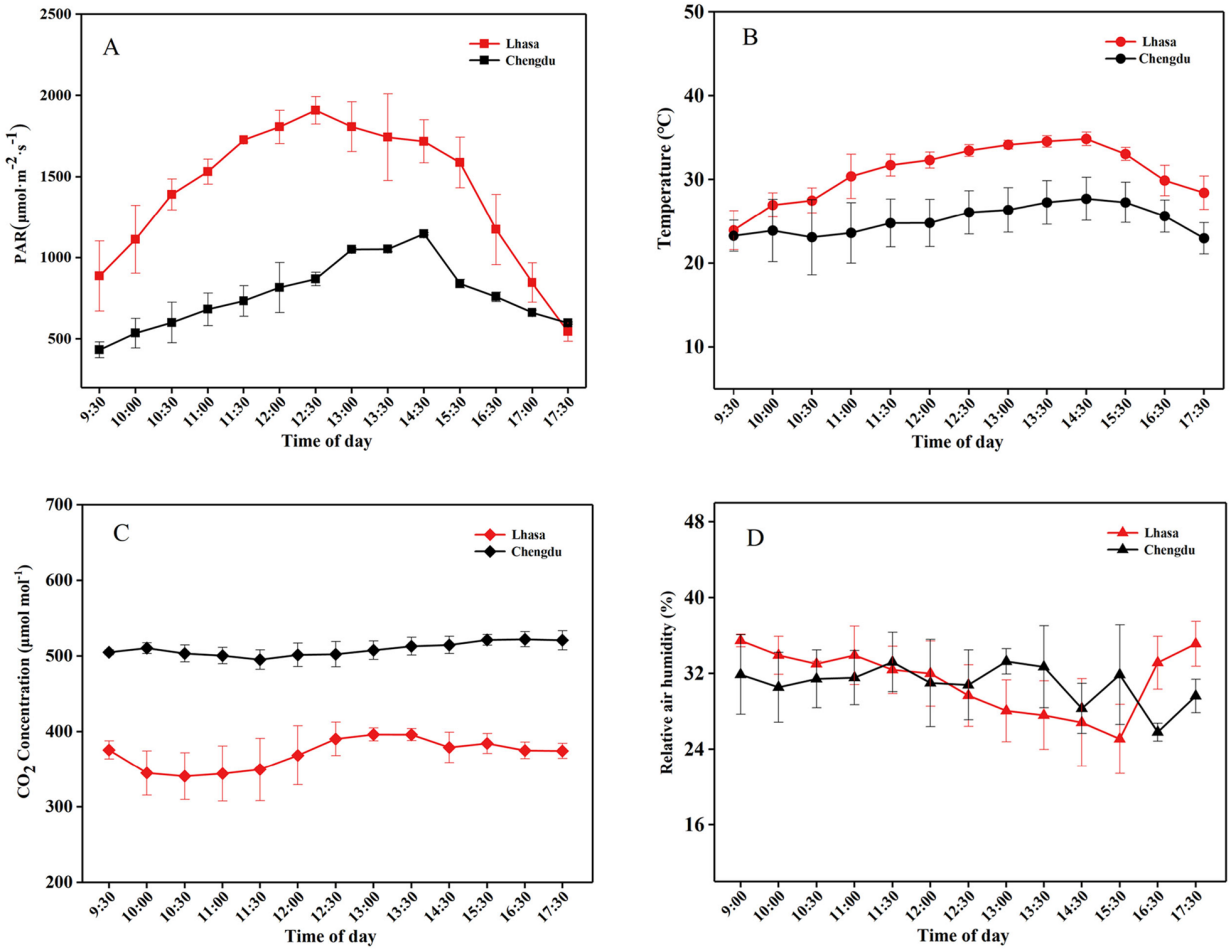
Daily variation curves of environmental factors in the experimental bases of the Academy of Agriculture and Animal Husbandry in Lhasa, Tibet, and the Chengdu rapeseed plantation, Sichuan University, China. **A** Diurnal variation of photosynthetically active radiation, **B** diurnal variation of ambient temperature, **C** diurnal variation of atmospheric relative humidity, **D** diurnal variation of atmospheric carbon dioxide concentration

Despite sharing a similar latitude, Lhasa and Chengdu exhibited obvious differences in various environmental factors, including sunlight intensity, temperature, humidity, and atmospheric CO_2_ concentration, due to their substantial altitude contrast of 4000 m (Fig. 2). During the rapeseed seedling stage, the photosynthetically active radiation (PAR) in Chengdu was significantly lower than in Lhasa. The average daily PAR in Lhasa was approximately 1413 μmol m^−2^ s^−1^, peaking at 1900 μmol m^−2^ s^−1^. Chengdu had an average daily PAR of 817 μmol m^−2^ s^−1^, with the peak at 14:30 and the maximum PAR of 1153.3 μmol m^−2^ s^−1^. The average daily temperature in Lhasa was around 30 °C, while Chengdu averaged 25 °C. Both regions had air humidity levels ranging between 30% and 40%. However, there was a significant difference in atmospheric CO_2_ concentration between the two regions. The average atmospheric CO_2_ concentration in the in Lhasa was less than 400 ppm, about two-thirds of the concentration in Chengdu.

### Identification of candidate rapeseed varieties with high photosynthetic efficiency

**Fig. 3:**
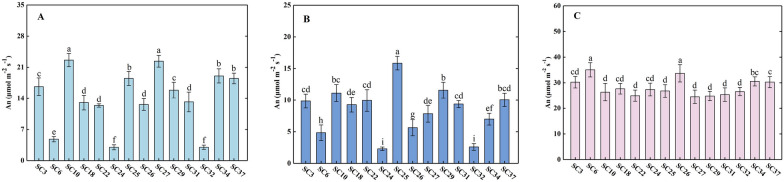
Net photosynthetic rate of different genotypes of *Brassica napus* at the following conditions **A** PAR:1500 μmol m^−2^ s^−1^, CO_2_: 300 μmol mol^−1^ **B** PAR: 1000 μmol m^−2^ s^−1^, CO_2_: 300 μmol mol^−1^ **C** PAR: 1500 μmol m^−2^ s^−1^, CO_2_: 500–600 μmol mol^−1^. Data shown are the means ± SE from three experimental replicates (*n* = 14, five plants per one experimental replicate). Different letters denote significant differences from multiple comparison test (*P* < 0.05) conducted for each measure condition

Different genotypes of *B. napus* exhibited significant differences in photosynthetic characteristics under different conditions (Additional file 1: Tables S1–S3). When the chamber light intensity was set to 1500 μmol m^−2^ s^−1^ and CO_2_ concentration was 300 μmol mol^−1^, the net photosynthesis rate (An) of *B. napus* leaves ranged from 3.01 to 22.63 μmol CO_2_ m^−2^ s^−1^. Among the 40 materials tested, SC3, SC10, SC25, SC27, SC34, and SC37 showed significantly higher photosynthetic efficiency compared to other materials, while SC6, SC24 and SC32 showed significantly lower photosynthetic efficiency (Fig. 3A). Under measurement conditions with the light intensity of 1000 μmolm^−2^ s^−1^ and CO_2_ concentration of 300 μmolmol^−1^, different genotypes of rapeseed showed leaf An values ranging from 2.27 to 16.11 μmol CO_2_ m^−2^ s^−1^. Among these materials, SC3, SC10, SC18, SC22, SC25, SC27, SC29, SC31 and SC37 showed higher leaf photosynthetic rate, while SC24 and SC32 had lower rate (Fig. 3B). When the chamber light intensity was set at 1500 μmol m^−2^ s^−1^ and CO_2_ concentration matched the atmospheric CO_2_ concentration (greater than 500 μmolmol^−1^), the photosynthetic rate of the leaves increased compared to the condition where CO_2_ concentration was 300 μmol mol^−1^, and the An ranged from 19.32 to 35.06 μmolCO_2_ m^−2^ s^−1^, with the photosynthetic rate of variety SC6 and SC26 being significantly higher than other varieties (Fig. 3C).

### Adaptation of rapeseed to plateau environment

#### Photosynthetic parameters, biomass and yield

**Fig. 4:**
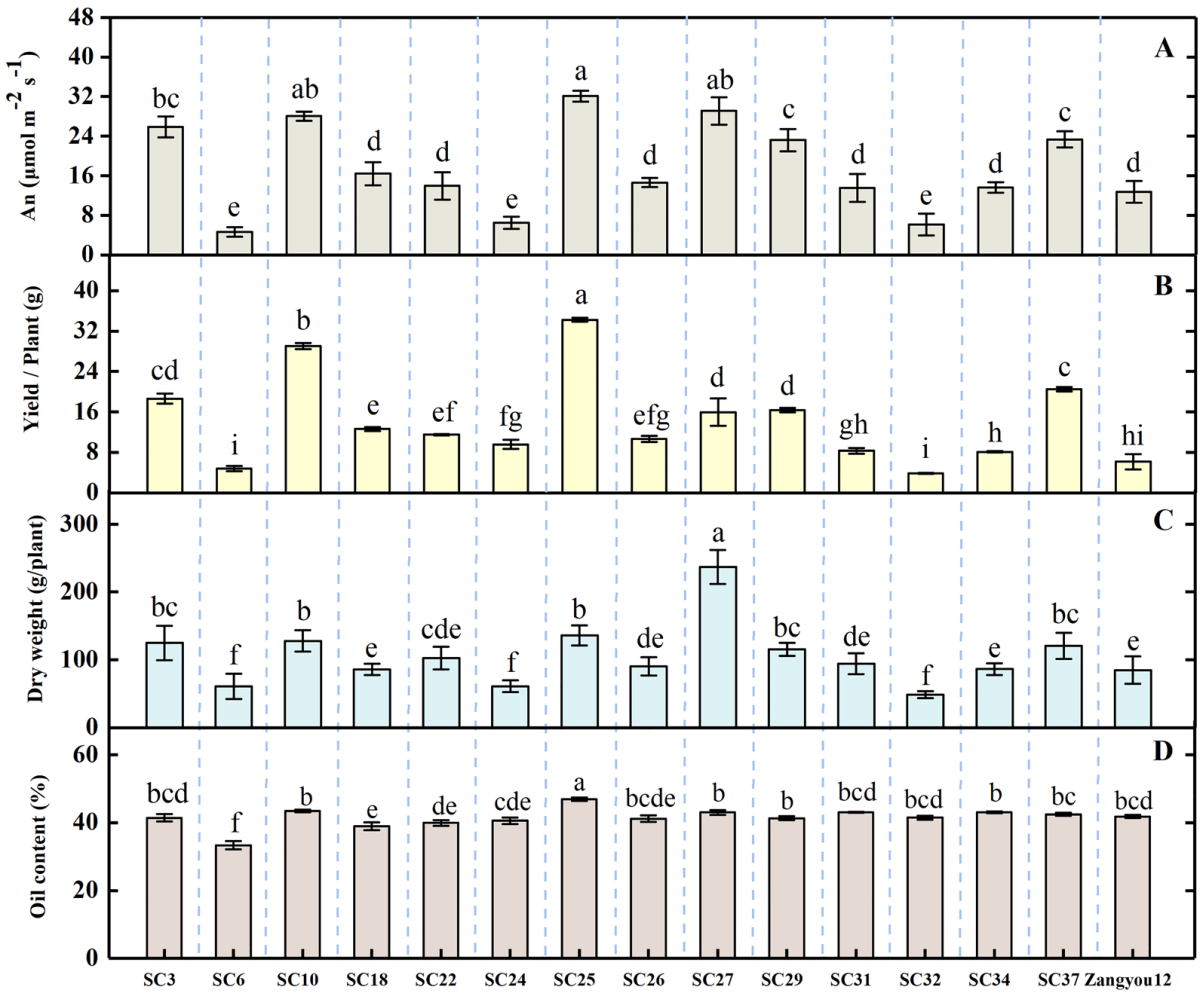
Differences in leaf net photosynthetic rate (**A**), yield per plant (**B**), biomass (**C**) and seed oil content (**D**) of various genotypes *Brassica napus* under plateau conditions in Tibet. Different letters indicate significant differences between different rapeseed genotypes. (*P* < 0.05)

To further confirm the adaptability of *B. napus* varieties with high photosynthetic efficiency to the plateau environment, we measured the photosynthetic parameters of these materials at the rapeseed cultivating base of the Lhasa Academy of Agricultural and Livestock Sciences (Additional file 1: Table S4). An of SC6, SC24 and SC32 leaves were lower under plateau environments, and An of SC6 was only 4.62 μmol CO_2_ m^−2^ s^−1^, while that of SC25 was 32.11 μmol CO_2_ m^−2^ s^−1^. Meanwhile, the An of SC3, SC10, SC25, SC27, SC29 and SC37 showed higher photosynthetic efficiency compared to Tibetan variety Zangyou12. (Fig. 4A).

In the plateau environment, high photosynthetic efficiency materials (SC3, SC10, SC25, SC27, SC29, and SC37) showed significantly higher yield per plant compared to other varieties, while there was no significant difference in yield per plant in the plain environment (Additional file 1: Table S5). Among them, the yield per plant of SC25 reached 34.25 g, approximately four times that of Zangyou12. (Fig. 4B). SC25 had 85% and 88.8% higher yield per plant than SC24 and SC32, respectively. SC27 had a grater biomass while SC6, SC24 and SC32 materials had less biomass than the other genotypes (Fig. 4C). Oil content is an important index for assessing the economic value of rapeseed. In the plateau environment, the oil content of SC3 seeds was 41.44%, SC25 was 46.87%, and SC6 was lower than other materials with only 33.31% (Fig. 4D). While in the Chengdu plain, there was no significant difference in seed oil content among them (Additional file 1: Table S5).

### Plant growth and agronomic traits

**Fig. 5:**
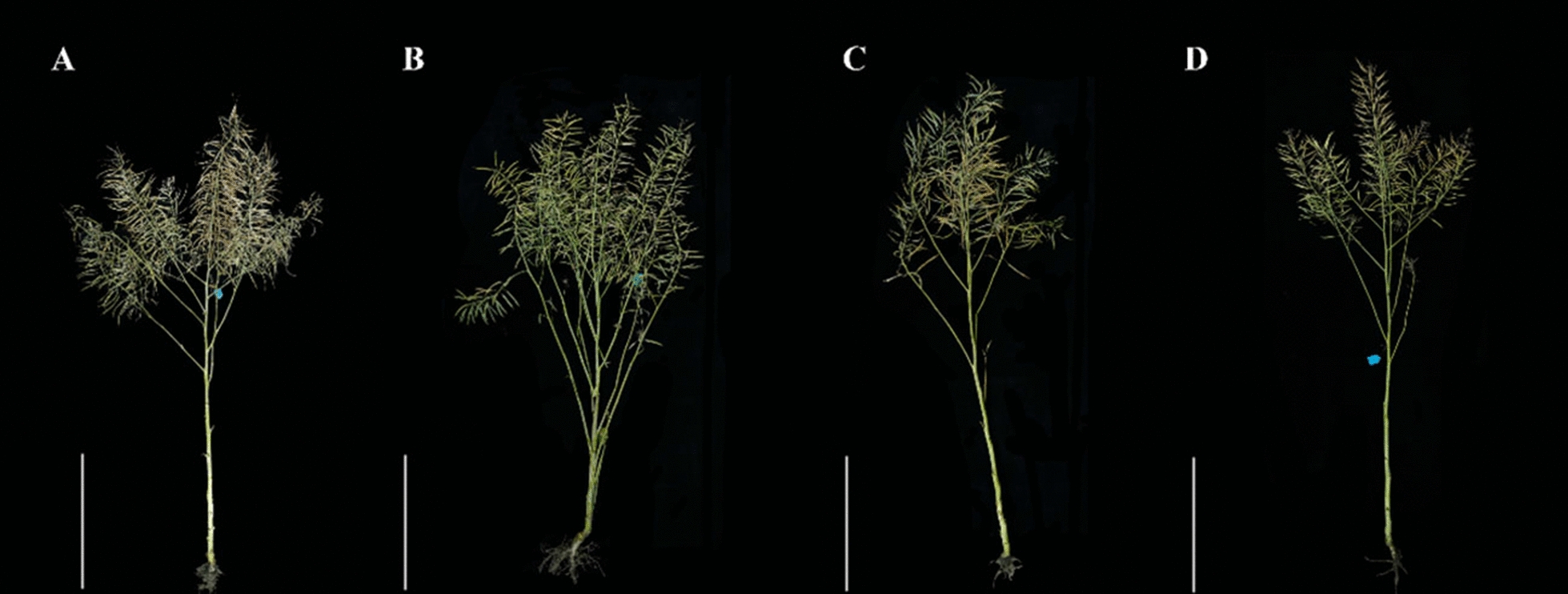
Different genotypes of *Brassica napus* were grown to maturity in the plateau environment (Lhasa, Tibet). **A** represents material SC10, **B** represents material SC25, **C** represents material SC32, and **D** represents material Zangyou12. Bars indicates 50 cm

Agronomic traits are statistically important in crop breeding. Therefore, observations of growth state (Fig. 5) and agronomic traits (Table 1) were conducted for different genotypes of *B. napus* cultivated in Tibet during the mature stage. Correlation analysis indicated a significant positive correlation between the An at the seedling stage and the number of siliques and yield per plant grown in Tibet. Moreover, the yield per plant had a significant positive correlation with the number of siliques per plant, branching number, and the number of seeds per silique (Table 2). SC3, SC10, SC29, and SC37 had greater plant height than others. In addition, SC3, SC25, and SC37 had more branches, and SC10, SC25, and SC37 had more siliques per plant. Material SC27 had the largest number of seeds per silique.

**Table 1:** Comparison of differences in agronomic traits: plant height, number of branches, number of siliques per plant and number of seeds per silique in different genotypes of *Brassica napus* under plateau conditions in Lhasa

Materia	Plant height (cm)	Branch number	Silique number per plant	Number of seeds per silique
SC3	150 ± 10.65 a	8.6 ± 1.14 a	333.6 ± 139.72 cd	13.00 ± 1.75 b
SC6	110.4 ± 10.64 def	5 ± 1.87 b	192 ± 90.06 e	9.34 ± 1.34 ef
SC10	148.4 ± 6.47 ab	7.2 ± 1.48 ab	417.6 ± 169.69 bc	10.38 ± 1.31 cde
SC18	108.2 ± 11.71 efg	6.2 ± 1.92 b	216.8 ± 167.84 d	12.38 ± 1.11 bc
SC24	105.8 ± 35.66 efg	5.2 ± 1.1 b	193 ± 89.18 e	13.44 ± 1.67 b
SC25	104.5 ± 11.68 fg	8.25 ± 1.26 a	667 ± 200.24 a	9.8 ± 2.49 de
SC26	91.33 ± 11.5 g	4.33 ± 0.58 b	235.33 ± 29.41d	13.17 ± 0.77 b
SC27	91.6 ± 5.32 g	4.6 ± 1.67 b	232.8 ± 60.78 d	15.42 ± 0.68 a
SC29	143.8 ± 16.71 abc	6.2 ± 3.11 b	234 ± 22.75 d	12.02 ± 1.67 bcd
SC31	127.6 ± 15.06 bcde	6.6 ± 2.3 b	259 ± 168.87 d	9.38 ± 0.85 ef
SC32	116.6 ± 10.04 def	5.2 ± 1.3 b	82.6 ± 32.05 e	7.42 ± 2.56 f
SC34	126.6 ± 6.99 cdef	6.8 ± 0.78 b	175.6 ± 19.5 e	12.94 ± 0.94 b
SC37	131.25 ± 9.22 abcd	7.5 ± 1.73 ab	566.75 ± 133.96 ab	10.55 ± 0.35 cde
Zangyou12	152 ± 16.51 a	6.5 ± 1 b	136.75 ± 32.83 e	10.5 ± 1.93 cde

Different letters indicate significant differences at the significant level (*P* < 0.05)

**Table 2:** Correlation analysis of agronomic traits, yield per plant and net photosynthetic rate (An) of seedling leaves of different genotypes of *Brassica napus* in the field

Agronomic traits	Plant height (cm)	Branch number	Silique number per plant	Number of seeds per silique	Yield per plant	An
Plant height (cm)	1					
Branch number	0.408^**^	1				
Silique number per plant	0.145	0.402^**^	1			
Number of seeds per silique	− 0.067	− 0.049	− 0.096	1		
Yield per plant	0.129	0.339^**^	0.94^**^	0.294*	1	
An	0.08	0.255^*^	0.47^**^	0.148	0.468^**^	1

^*^Indicates significant correlation at the level of *P* < 0.05)

^**^Indicates significant correlation at the level of *P* < 0.01

### Differences in fatty acid content and composition

**Fig. 6:**
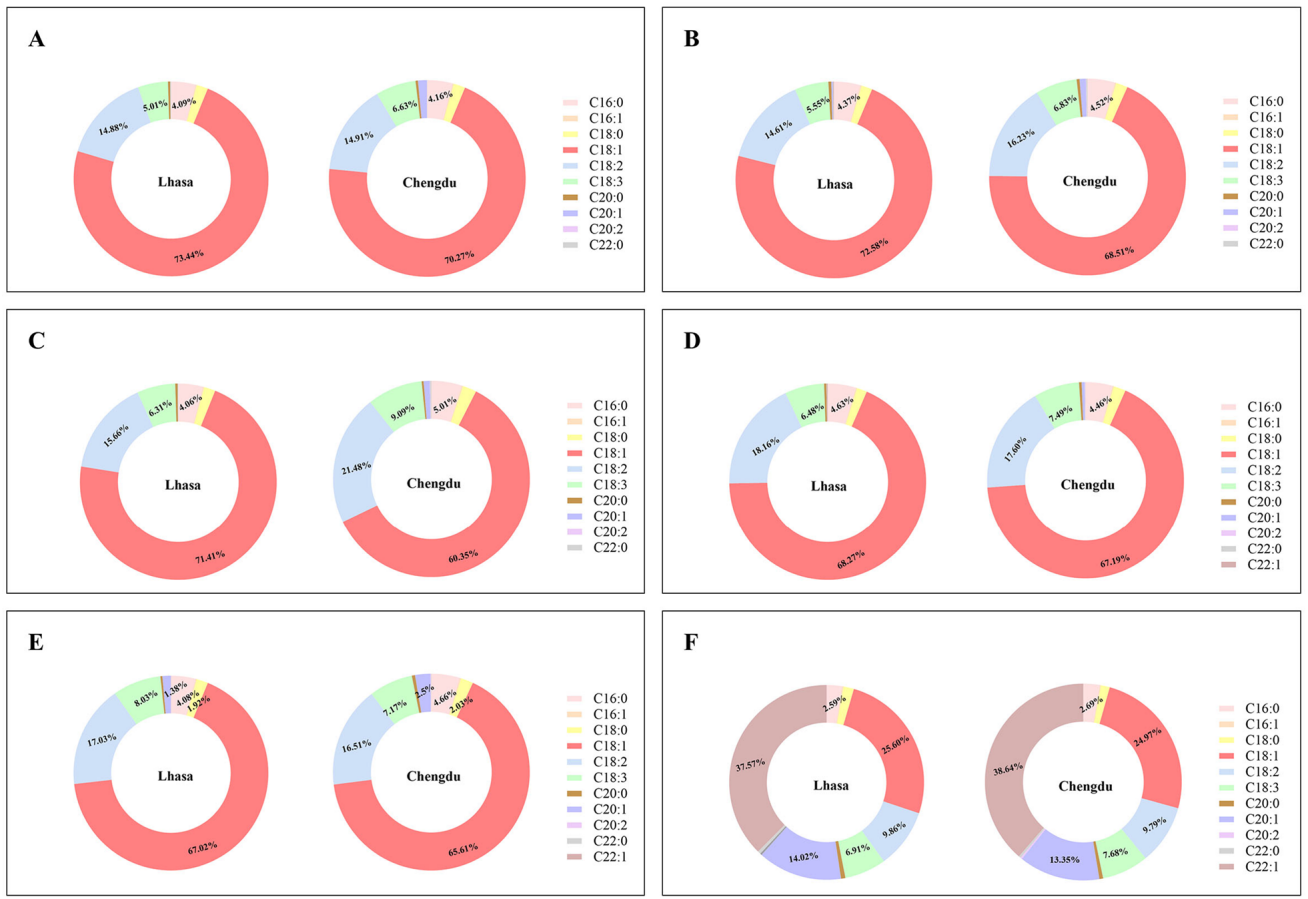
Fatty acid composition in seeds of different rapeseed varieties in two ecological environments: plain (Chengdu, Sichuan) and plateau (Lhasa, Tibet). **A**–**F** represent the fatty acid composition of *Brassica napus* from materials SC3, SC10, SC25, SC27, SC37 and SC32, respectively

Fatty acids constitute the primary components of rapeseed oils, with oleic acid (C_18_H_34_O_2_), linoleic acid (C_18_H_32_O_2_) and erucic acid (C_22_H_42_O_2_) serving as quality indicators. In the plateau environment, the oleic acid content of *B. napus* varieties SC3, SC10, SC25, SC27 and SC37 ranged from 67.02% to 73.44%, while in the Chengdu Plain it ranged from 60.35% to 70.27%. Therefore, the oleic acid content of rapeseed increased in plateau (Additional file 1: Table S6). The linoleic acid content increased in SC27 and SC37 under plateau conditions, while SC3, SC10 and SC25 decreased. The erucic acid content of high erucic acid material SC32 was 37.57% in the plateau and 38.64% in the plain. (Fig. 6).

### Photosynthetic carbon metabolism

**Fig. 7:**
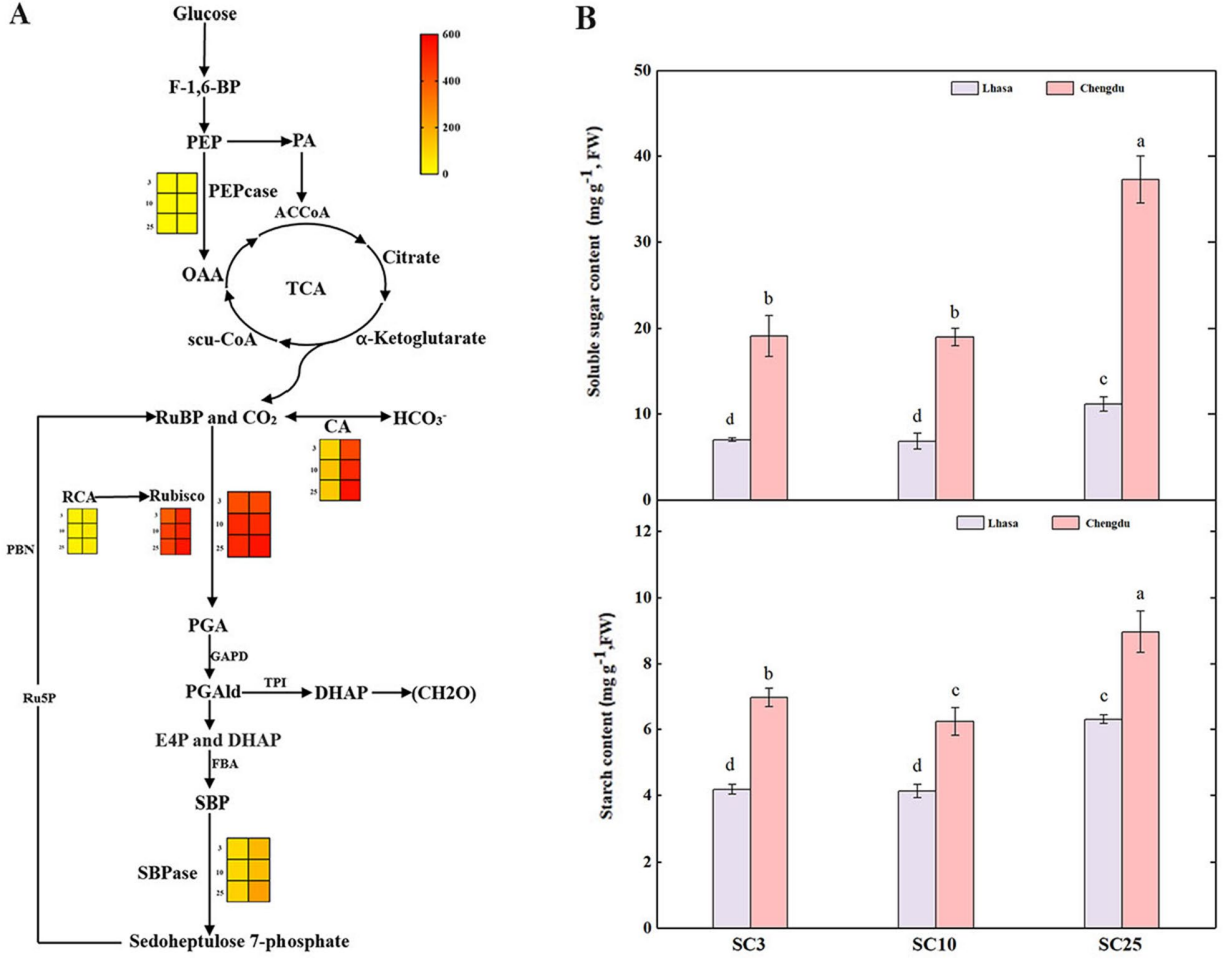
Enzyme activities (**A**, the left side of the heat map shows Lhasa, the right side shows Chengdu) and photosynthetic metabolite contents (**B**) related to photosynthetic carbon transfer in seedling leaves of two ecological environments of *Brassica napus* varieties: SC3, SC10, and SC25 in plain (Chengdu, Sichuan) and plateau (Lhasa, Tibet). Different letters indicate significant differences between different genotypes of rapeseed. (*P* < 0.05)

Once the light intensity reaches the leaves’ saturation point, their photosynthetic rate no longer increases. At this stage, factors such as electron transfer reactions, Rubisco activity, phosphoenolpyruvate (PEP) metabolism become limiting factors. Rubisco, RuBPcase, SBPase, RCA, CA and PEPcase are all critical enzymes in the dark reactions of photosynthesis. In plateau conditions, the activities of Rubisco, CA, SBPase, and RCA in *B. napus* leaves were significantly lower than those in the plain. The carboxylation activity of Rubisco and PEPcase increased in plateau, but the differences were not significant. (Fig. 7A).

Changes in environmental factors can have an impact on carbon metabolism in leaves, resulting in changes in the carbohydrate content. In Lhasa, SC3, SC10 and SC25 showed a decrease in soluble sugar and starch content. Under the same environmental conditions, various rapeseed genotypes exhibited different leaf carbohydrate content, with material SC25 showing a higher concentration of photosynthetic products than SC3 and SC10. (Fig. 7B).

## Discussion

Photosynthesis is the physiological basis for crop yield, and the strength of light energy utilization determines the crop yield [28]. The selection and development of rapeseed varieties with high photosynthetic efficiency to enhance light energy utilization are crucial for increasing production. When CO_2_ concentration is lower than two-thirds of the normal atmospheric CO_2_ concentration, it affects plant growth [29]. The experimental base of the Lhasa Academy of Agricultural and Animal Husbandry Sciences is seven times higher in altitude than the Chengdu Plain at the same latitude. It has thin air, intense photosynthetically active radiation, and an atmospheric CO_2_ concentration about two-thirds that of the Chengdu Plain.

Both light intensity and CO_2_ concentration affects the efficiency of light energy utilization in crops. This study showed that different genotypes of rapeseed seedlings respond differently to changes in light intensity and CO_2_ concentration. *B. napus* varieties SC3, SC10, SC25, SC27, and SC37, showed high photosynthetic efficiency when subjected to high light intensity (PAR of 1500 μmolm^−2^ s^−1^) and low CO_2_ concentration (300 μmol mol^−1^) conditions. In addition, these varieties exhibited significantly higher net photosynthesis rate (An), yield per plant, and biomass compared to others in plateau. Interestingly, SC6 and SC26 with high photosynthetic efficiency under high light intensity conditions (PAR of 1500 μmolm^−2^ s^−1^) were not dominant in the plateau phenotype. This indicates that low CO_2_ concentration is the key environmental factor limiting rapeseed growth in the plateau. On the plateau, the An of rapeseed leaves at the seedling stage are lower than the results observed under high light intensity (PAR of 1500 μmol m^−2^ s^−1^) and atmospheric CO_2_ concentration conditions in the plain. This also indicates that the low CO_2_ concentration limits the photosynthesis of rapeseed leaves when light intensity reaches saturation on the plateau. Therefore, increasing field CO_2_ concentration through agricultural production measures in plateau can enhance photosynthetic efficiency and increase rapeseed production.

There is a correlation between the photosynthetic rate and the morphological structure of plants. Biomass and agronomic traits are important indicators of crop growth. Studies have shown a significant positive correlation between soybean yield and leaf net photosynthetic rate [30], but some research shows that the photosynthetic rate of corn is unrelated to productivity, and selecting corn varieties with high photosynthetic rate may even lead to reduced productivity [31]. This study found that the high photosynthetic efficiency materials SC3, SC10, SC25, SC29 and SC37 in plateau environment had significantly higher yield per plant, biomass, and oil content compared to others. Correlation analysis also indicates a significant positive correlation between An and the yield per plant of rapeseed. Therefore, it shows that high photosynthetic efficiency rapeseed genotypes have a certain yield potential, consistent with previous studies [32]. Breeding rapeseed materials with high photosynthetic efficiency adapted to low CO_2_ concentration is crucial for improving rapeseed production and quality in plateau regions.

**Fig. 8:**
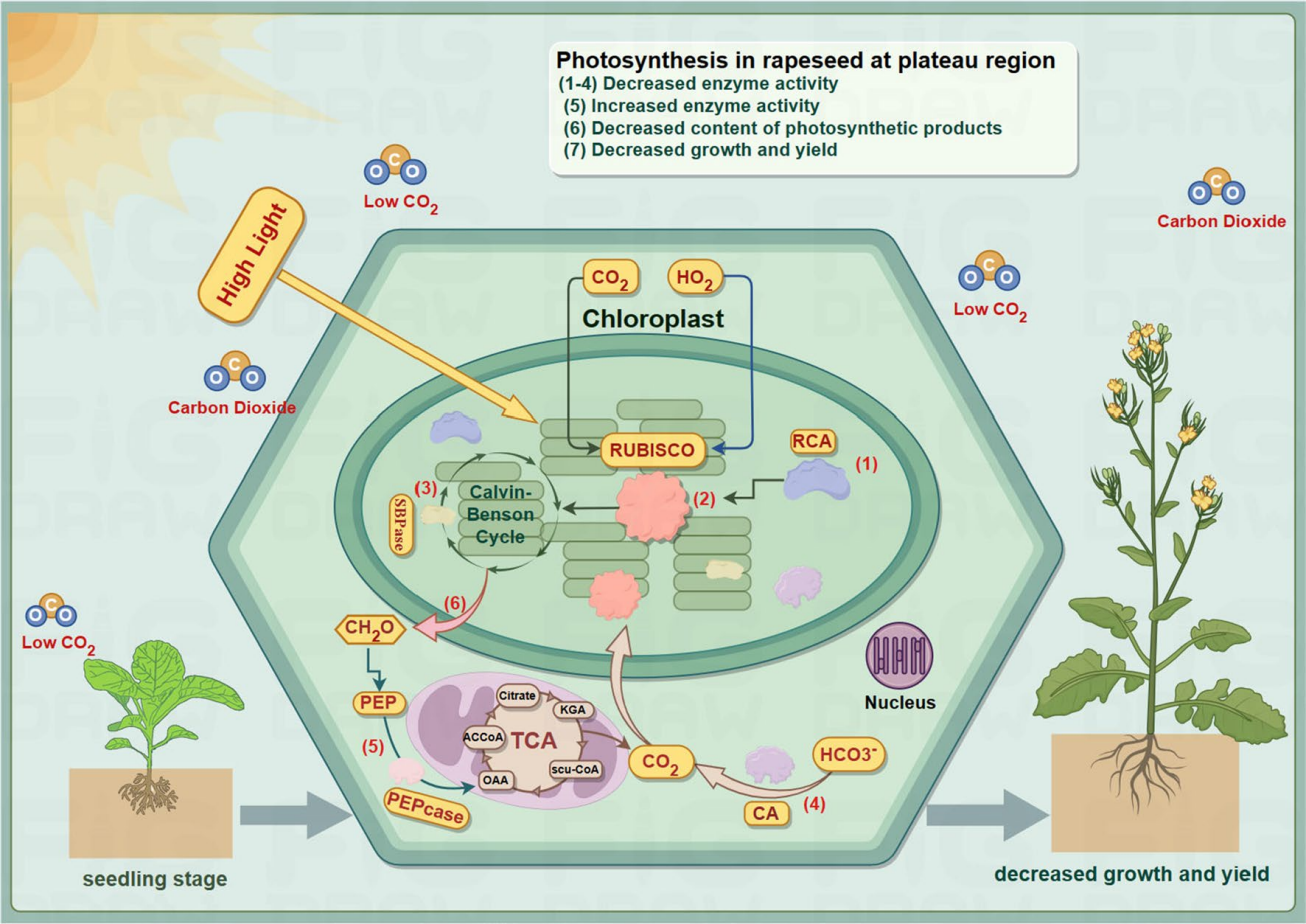
Mechanisms of changes in leaf photosynthesis at physiological and biochemical levels in rapeseed under plateau environment. The pattern diagrams (1) Rubisco activate (RCA), (2) carboxylation activity of Rubisco, (3) Sedoheptulose-1,7-bisphosphatase (SBPase), (4) Carbonic Anhydrase(CA) (5) Phosphoenolpyruvate carboxylase (PEPcase), and (6) represent photosynthetic product formation

The carbon cycle of photosynthesis involves CO_2_ fixation, photosynthetic product allocation, and carbon metabolism, regulated by multiple enzymes, which significantly impact crop yield, growth, and adaptability. Under stress conditions, the activity of PEPcase increases in C_3_ plants [33]. The results of this study show that under plateau conditions, the activities of Rubisco, CA, SBPase and RCA are reduced in SC3, SC10 and SC25. This reduction may result from low CO_2_ concentration, limiting the rate of photosynthesis, causing insufficient energy and carbon supply and the activation of negative regulatory mechanisms. Due to the intense light and low CO_2_ concentration in the plateau environment, rapeseed leaves, upon reaching light saturation, undergo continuous carbon reactions, consuming ATP generated in the light reactions. Consequently, the carboxylation activity of Rubisco increases in the plateau environment. The low CO_2_ concentration can reduce the synthesis of photosynthetic products in plant leaves [34], which is consistent with the results of our study. Under plateau conditions, SC3, SC10, and SC25 have significantly lower soluble sugar and starch contents in their leaves compared to the Chengdu Plain. These differences may be related to the decreased photosynthesis rate of the plants. Therefore, exploring the key pathway of photosynthesis in rapeseed may help to elucidate the molecular mechanisms of C_3_ plant adaptation to plateau region (Fig. 8).

Rapeseed is one of the important sources of vegetable oil, and its fatty acid composition directly impacts the quality and nutritional value of the oil. Previous research has found that light intensity affects seed oil content and fatty acid synthesis. In this study, we found that under plateau conditions, the oleic acid content increased, and the erucic acid content of high-erucic acid rapeseed decreased. High light intensity provided a large amount of ATP and NADPH for fatty acid synthesis in plastids, favoring oleic acid synthesis [35]. This could be due to the low atmospheric CO_2_ concentration, which affects the carbon metabolism of rapeseed, leading to changes in fatty acid metabolism and synthesis. This offers a new perspective for the breeding and design of industrial rapeseed.

## Conclusion

During the cultivation period of rapeseed on the plateau (Lhasa, Tibet), sunlight intensity is approximately double that of the plain (Chengdu, Sichuan), and atmospheric CO_2_ concentration is about two-thirds of that in the plain. The rapeseed materials SC3, SC10, SC25, SC27, SC29 and SC37 with high photosynthetic efficiency under low CO_2_ concentrations rather than high light intensity, exhibited significant advantages in biomass, yield, and oil content when cultivated on the plateau, indicating that the CO_2_ is the key environmental factor which limited rapeseed production in plateau. Simultaneously, PEPcase activity in rapeseed leaves increased at plateau, while SBPase and RCA activities decreased, and soluble sugar and starch contents also decreased. The oleic acid content in rapeseed increased, while the erucic acid content exhibited a decreasing trend. In conclusion, we found that both the key enzyme activities of carbon reactions and carbon metabolism levels in rapeseed are reduced in plateau environment. The key environmental factor limiting plateau rapeseed production is the low atmospheric CO_2_ concentration. Also, the rapeseed materials SC3, SC10, SC25, SC27, SC29, and SC37 exhibit the higher adaptabilities and breeding values in plateau environments. Hence, our study provides the theoretical and practical foundations for plateau-adapted rapeseed breeding.

## Materials and methods

### Plant material and growth conditions

40 *Brassica napus* varieties (designated as SC1–40) were used as experimental materials. Seeds that were plump and uniform in size were selected and treated with 10% H_2_O_2_ for 30 min for surface sterilization. After wash with sterile water for 2–3 times, the seeds were placed on two layers of moistened filter paper in Petri dishes for germination under a 14-h light/10-h dark photoperiod at 28 °C in the growth chamber. After seed germination, they were transplanted into pots (14 cm × 13 cm) filled with nutrient soil, with 2 seedlings per pot, and were cultivated in the plant culture room (located at 104°06′E, 30°67′N, and 500 m above sea level). Field experiments were conducted at the experimental base of the Tibet Lhasa Academy of Agricultural and Animal Husbandry Sciences, located at coordinates (91°06′E, 29°36′N), at an altitude of 3650 m above sea level. The experimental plots were arranged in a completely randomized block design with three replications of the plots, with each plot measuring 1.8 m^2^ (6 × 0.3 m), consisting of 6 rows with a row spacing of 0.3 m and a plant spacing of 0.16 m. Standard field management practices were followed uniformly.

### Photosynthetic leaf gas exchange measurements

Photosynthetic parameters of *B. napus* at the six-leaf stage were assessed using a portable photosynthesis measurement instrument, Li-6400XT (Li-Cor, Lincoln, NE, USA), with a leaf chamber size of 2 × 3 cm^2^. The red–blue light source, 6400-02B, was employed, with a leaf chamber temperature set at 28 °C. Measurements were conducted under the following conditions: (1) the leaf chamber light intensity was set at 1500 μmol m^−2^ s^−1^, and the chamber’s CO_2_ concentration at the ambient atmospheric CO_2_ concentration. (2) The leaf chamber light intensity was set at 1500 μmol m^−2^ s^−1^, and the CO_2_ concentration in the chamber was adjusted to 300 μmol mol^−1^. (3) The leaf chamber light intensity was set at 1000 μmol m^−2^ s^−1^, and the chamber’s CO_2_ concentration was set at 300 μmol mol^−1^. Under given conditions, photosynthetic parameters of the sixth leaf of 40 rapeseed materials were measured, including net photosynthesis rate (An; μmol m^−2^ s^−1^), intercellular CO_2_ concentration (Ci; μmol mol^−1^), transpiration rate (Tr; mmol m^−2^ s^−1^), and stomatal conductance (Gs; mmol m^−2^ s^−1^). This study measured the photosynthetic characteristics of *B. napus* leaves in the plateau region of Tibet. Random selection of five plants with similar growth from each experimental plot was conducted. The temperature inside the leaf chamber was maintained at a constant 28 °C, while the light intensity and CO_2_ concentration were consistent with the natural environmental conditions outside.

### Plant growth and yields

Five plants per plot were randomly selected at the maturity stage of *B. napus*, and biomass, field agronomic traits and seed oil content were measured. Field agronomic traits indicators comprised plant height, number of branches, number of effective siliques per plant, number of seeds per silique and yield per plant.

### Leaf carbon metabolism physiological indicators

The enzyme activities of the key enzymes involved in photosynthetic carbon reactions, namely, RuBPcase, PEPCase, RCA and SBPase, were measured. Samples of fresh leaf tissue(0.1 g)were extracted using a tissue homogenizer with 900 μL phosphate buffer (pH = 7.2–7.4, concentration of 0.01 mol/L) at 4 ℃ The resulting sample solution was diluted fivefold with sample diluent and added into the enzyme plate for an incubation at 37 ℃ for 30 min followed by 5 times washing with detergent. Then, except for the blank wells, each well was supplemented with enzyme reagent for incubation at 37 ℃ for 30 min, followed by 5 washes at the end of the reaction. Subsequently, chromogenic reagent was added to the plate and allowed to develop color at 37 ℃ for 10 min. The reaction was then terminated using the termination solution. Finally, the absorbance value was determined by setting the wavelength at 450 nm using a Microplate Reader, and the zero of the blank wells was adjusted accordingly.

The soluble sugar and starch contents were determined by anthrone colourimetry. Oil content and fatty acid content of rapeseed were determined using a standard benchtop NIR spectrometer (DS 2500, Foss).

### Statistical analysis

The data were statistically analyzed using SPSS 21.0 (SPSS Inc., Chicago, IL, USA), and Origin 9.0 (OriginLab Corporation, Northampton, MA, USA) was used for graphical presentation of data. Pattern drawing by Figdraw website.

### Supplementary Information

**Additional file 1: Table S1.** Photosynthetic parameters of rapeseed varieties at 1500 μmol m^−2^s^−1^ photosynthetically active radiation and 300 μmol mol^−^^1^ CO_2_ concentration. **Table S2.** Photosynthetic parameters of rapeseed varieties at 1000 μmol m^−2^s^−1^ photosynthetically active radiation and 300 μmol mol^−^^1^ CO_2_ concentration. **Table S3.** Photosynthetic parameters of rapeseed varieties at 1500 μmol m^−2^s^−1^ photosynthetically active radiation. **Table S4.** Photosynthetic parameters among rapeseed varieties in a plateau environment. *An* net photosynthetic rate, *Gs* stomatal conductance, *Ci* intercellular carbon dioxide concentration, *Tr* transpiration rate. **Table S5.** Oil content and yield in seeds of different rapeseed varieties in two ecological environments: plain (Chengdu, Sichuan) and plateau (Lhasa, Tibet). **Table S6.** Fatty acid composition in seeds of different rapeseed varieties in two ecological environments: plain (Chengdu, Sichuan) and plateau (Lhasa, Tibet).

## Data Availability

Data for the results of this study are available from the paper and its Additional Information files.
